# Production of Gamma-Aminobutyric Acid by *Levilactobacillus brevis* AK19B: Strain Characterization and Process Optimization Using Box–Behnken Design

**DOI:** 10.1007/s12602-026-10965-4

**Published:** 2026-03-12

**Authors:** Habibe Memiş, Aybike Kamiloğlu

**Affiliations:** Faculty of Engineering, Food Engineering Department, Bayburt University, Bayburt, 69000 Turkey

**Keywords:** *Levilactobacillus brevis*, GABA, Optimization, Box–Behnken design, Techno-functional properties

## Abstract

*Levilactobacillus brevis* AK19B strain demonstrated promising probiotic potential as a hydrophilic lactic acid bacterium with valuable phytase activity. It also exhibited strong antioxidant properties and maintained viability in bile salt-rich environments, suggesting robustness in gastrointestinal conditions. To maximize gamma-aminobutyric acid (GABA) production, a Box–Behnken design was applied to assess the effects of monosodium glutamate (MSG) concentration, NaCl concentration, pH, and temperature. The results showed that these factors significantly influenced GABA synthesis, with their interactions well-modeled by a quadratic response surface. Optimal conditions were identified as pH 5.136, MSG concentration of 297 mM, temperature at 26 °C, and NaCl concentration of 2.82%, under which the predicted GABA production reached 43.85 mg/mL. These findings highlight AK19B as a promising candidate for functional food applications and industrial GABA biosynthesis.

## Introduction

Gamma-aminobutyric acid (GABA), a four-carbon non-protein amino acid, plays a pivotal role as an inhibitory neurotransmitter in the mammalian central nervous system [1]. Its therapeutic promise is well-documented: from alleviating symptoms of anxiety and depression to supporting cerebral energy metabolism and enhancing physiological resilience during stress and hypoxia [2]. GABA also exhibits anticancer potential by suppressing proliferation in various cancer cell lines, particularly those derived from colorectal, mammary gland, and hepatic tissues [3]. Additionally, it contributes to immune modulation [4] and supports improved sleep quality and relaxation [5]. In plants, both active growth stages and germination phases are characterized by significant GABA accumulation, highlighting its fundamental role in plant physiology [6].

Within microbial ecosystems, lactic acid bacteria (LAB) stand out as efficient and safe GABA producers, underscored by their GRAS (Generally Recognized As Safe) status [7]. GABA biosynthesis in these organisms centers on the glutamate decarboxylase (GAD) system comprising either *gadA* or *gadB* genes and the *gadC* antiporter [8]. LAB are frequently sourced from traditionally fermented foods, plant-based fermentations, and gastrointestinal tracts, with high-performing strains isolated from products such as cheese, yogurt, kimchi, paocai, bread, fermented fish products, and sausages [7, 9, 10].

Multiple environmental and nutritional factors affect GABA production efficiency, including pH, temperature, MSG concentration, NaCl levels, and cofactor availability (e.g., PLP) [11, 12]. Acidic environments enhance GAD activity and facilitate cellular pH homeostasis during decarboxylation [13, 14]. Due to limited endogenous glutamate biosynthesis in LAB, supplementation with L-glutamic acid or MSG is typically required for optimal GABA yields [8, 15]. Moreover, additives like chloride ions, arginine, and malate have also been shown to increase GABA yield [16–18].

However, most studies tend to examine GABA production independently, often neglecting potential synergies between GABA synthesis and other beneficial enzymatic traits such as phytase activity, antioxidant capacity, or salt tolerance. This segmented focus poses the risk of overlooking multifunctional strains with greater commercial and nutritional value. Sourdough ecosystems, characterized by acid stress and complex microbial interactions, offer a promising field for isolating LAB with both robust GAD functionality and stress resilience [19–21].

In light of this, the current study aimed to isolate and characterize a novel *Levilactobacillus brevis* strain (AK19B) from sourdough, investigating its capacity for GABA production alongside phytase activity, antioxidant potential, and stress tolerance (e.g., against salt and oxidative challenges). By employing a Box–Behnken experimental design, we optimized GABA synthesis in a manner that preserved these multifunctional traits, modeling the interactive influences of MSG concentration, pH, NaCl, and temperature on both primary and secondary metabolite production. This comprehensive strategy offers novel mechanistic insight into the multifunctionality of *L. brevis* AK19B and its promise as an advanced probiotic capable of simultaneously enhancing nutrient bioavailability and bioactive compound production. This study differs from existing studies in the literature in that it evaluates the phytase activity, antioxidant capacity, and probiotic properties of the Levilactobacillus brevis AK19B strain alongside its GABA production within the same experimental design. While previous studies have generally focused solely on increasing GABA production yield [19, 20], this study simultaneously examined multiple bioactive properties of the strain, thus providing a more comprehensive functional evaluation [7, 9]. This approach contributes to the identification of strains with multifaceted metabolic potential and offers a new perspective for functional food applications.

## Material and Method

### Identification of Isolates Capable of Producing GABA

In this study, active cultures were used to determine the GABA production capabilities of 30 sourdough-derived LAB strains, which were incubated for 96 h at 37 ℃ in de Man, Rogosa and Sharpe (MRS) broth [22] containing 53 mM monosodium glutamate. After centrifugation at 5000 rpm for 15 min, the supernatant was stored for the determination of GABA production. Supernatant fractions were evaluated using thin layer chromatography (TLC). Monosodium glutamate and GABA standards and supernatants belonging to the strains were dropped on the TLC layer with a volume of 2 µl. Afterwards, it was immersed in a medium containing butanol: acetic acid: distilled water (4:1:1) and left for 2 h for fraction separation. After spraying with 0.4% ninhydrin solution in ethanol, the TLC plate was heated at 100 °C for 5 min to visualize the amino acid derivatives.

### Identification of LAB

#### Molecular Identification

Molecular characterization of the bacterial strain was performed using the genomic DNA isolation kit (EcoTech-EcoPURE Genomic DNA Kit) within the framework of the protocol given below.

####  16s rRNA Analyses

PCR plan: 25 µL Master mix (DNA polymerase, dNTPs, optimized buffer), 2 µL Forward primer 10 µM, 2 µL Reverse primer 10 µM, 10 pg-50 µg Template DNA, made up to 50 µL with PCR (Polymerase Chain Reaction) program: Denaturation at 95 °C for 2 min, 36 cycles, denaturation at 94 °C for 1 min, binding at 53 °C for 1 min, and elongation at 72 °C for 2 min. Finally, the reaction was carried out in a PCR thermal cycler with an elongation step at 72 °C for 5 min [23].

Sequence analysis of the obtained PCR product was performed with BMLabosis (ANKARA). BioEdit 7.2 program was used to evaluate the analysis results.

#### Determination of GABA Production Amounts by HPLC

GABA content was analyzed at a wavelength of 295–500 nm using an HPLC (Shimadzu) system equipped with an Inertsil ODS 3 column (GL science, 5 µm, 4.6 × 250 mm). Solvent A (6.25 mL acetic acid, 1.97 g sodium acetate, 200 mL acetonitrile, 1 L ultrapure water) and solvent B (acetonitrile) were used as mobile phase (1:1, v/v). Samples of 1 µL were injected. Separation was achieved at a flow rate of 1 mL/min at a column temperature of 25 °C. With the derivatization process, the separation of the components is ensured with peaks formed in different places at different times in the HPLC by changing the molecules. For this purpose, after mixing 100 µL of sample, 100 µL of DCI (50 mg dansyl chloride, 10 ml acetone) and 800 µL of borate: NaOH (0.2 M Na_2_B_4_O_7_.10 H_2_O pH 9.3) were added and treated with ultrasound for 30 min at 40 °C. Then, 500 µL of the clear liquid obtained at the end of the period was placed into the HPLC vial without shaking, and 500 µL of methanol was added to make it ready for analysis. The photon diode array due to derivatized GABA was detected by the fluorescence detector. GABA identification was made by comparing the retention times of GABA (Sigma-Aldrich) used as a standard with the retention times of the samples. In order to determine the GABA concentrations in the samples, the GABA level in the supernatant of the LAB strain was determined by testing the standards prepared at different concentrations (0.01–0.1–0.25–0.50–1.0 mg/mL) [24].

### Determination of Techno-functional Properties of Lactic Acid Bacteria

#### Growth at Different Ph Value

MRS broth with different pH values (pH 4, 5, 8) was prepared to examine the growth of the isolate under varying pH conditions. The initial pH of the culture medium was adjusted using 1 N NaOH or 1 N HCl prior to sterilization. For each pH condition, 10 mL of the adjusted MRS broth was transferred into sterile 50 mL screw-capped polypropylene centrifuge tubes.

A 100 µL aliquot of a 24-h culture of *Levilactobacillus brevis* AK19B, previously grown in MRS broth at 30 °C under static conditions, was inoculated into each tube. The inoculated media were then incubated at 30 °C for 24 h under stationary (non-shaking) conditions to simulate anaerobic fermentation-like environments. At the end of the incubation, the effect of pH on microbial growth was determined by measuring the absorbance at 600 nm against a blank using a UV–Vis spectrophotometer (UV-1800, Shimadzu Corporation, Kyoto, Japan) as described by Sifeeldein et al. [25]. The growth was categorized as poor (≤ 1.0), good (1.1–1.5), or very good (≥ 1.5) based on absorbance values.

#### Growth at Different Temperatures

To examine the growth of the strain under different temperature conditions, 5 mL of MRS broth was dispensed into sterile 15 mL screw-capped polypropylene culture tubes. The medium was sterilized, and 0.1 mL of a 24-h culture of *Levilactobacillus brevis* AK19B was inoculated into each tube. Inoculated cultures were incubated under static (non-shaking) conditions at three different temperatures: 4 °C for 7 days, 25 °C for 2 days, and 45 °C for 3 days, respectively. These time intervals were selected to allow visible growth at each temperature, accounting for differences in metabolic rate. At the end of each incubation period, growth was quantified by measuring absorbance at 600 nm against a blank using a UV–visible spectrophotometer (UV-1800, Shimadzu Corporation, Kyoto, Japan), following the method of Drosinos et al. [26]. Growth was categorized based on absorbance as poor (≤ 1.0), good (1.1–1.5), or very good (≥ 1.5).

#### Growth at High Salt Concentration

To evaluate the strain’s resistance to high salt concentrations, *Levilactobacillus brevis* AK19B was cultured in MRS broth supplemented with NaCl at various concentrations (7, 10 and 15%). For each condition, 5 mL of MRS broth was dispensed into sterile 15 mL screw-capped polypropylene tubes. Each tube was inoculated with 0.1 mL of a 24-h pre-culture, previously grown at 37 °C in MRS broth under static conditions. The inoculated media were then incubated at 37 °C for 48 h without agitation (i.e., under static conditions) to simulate gastrointestinal temperature conditions. Growth was assessed by measuring optical density at 600 nm using a UV–visible spectrophotometer (UV-1800, Shimadzu Corporation, Kyoto, Japan). The results were interpreted based on absorbance values as follows: poor (≤ 1.0), good (1.1–1.5), and very good (≥ 1.5).

#### Thermotolerant Capacity

The thermotolerance of the isolated *Levilactobacillus brevis* AK19B strain was evaluated according to the method of Pérez-Chabela et al. [27], with slight modifications. Initially, the strain was propagated in 5 mL of MRS broth contained in sterile 15 mL screw-capped polypropylene tubes and incubated at 37 °C for 24 ± 2 h under static (non-shaking) conditions. The optical density (OD) of the culture was monitored spectrophotometrically at 600 nm, and cultures with OD ≈ 1.0 were considered ready for thermal challenge. The cultures were then exposed to 70 °C in a water bath for 60 min. Following heat treatment, 1 mL aliquots were aseptically withdrawn and plated on MRS agar. The plates were incubated at 37 °C for 24 h under aerobic conditions. Strains exhibiting viable growth greater than 300 colony-forming units per mL (cfu/mL) after incubation were considered thermotolerant.

#### Resistance to Bile Salt

In order to evaluate the low pH and bile salt resistance, the LAB strain was grown overnight and then washed twice with 0.85% physiological saline. All strains were then set to 1 at OD600 nm. For control purposes, cultures were inoculated into MRS broth at a rate of 1% (v/v) of the 24-h pre-culture without any pH adjustment or bile salt addition. Likewise, 1% (v/v) of the same pre-culture was inoculated into MRS broth supplemented with 0.3% (w/v) bile salts (bovine bile, Sigma-Aldrich) and adjusted to pH 4 using 1 M HCl. These processes were carried out in a glass tube and incubated at 37 °C without shaking. After incubation, OD600 measurements were made in the spectrophotometer (UV-1800, UV Spectrophometer, Shimadzu Corporation, Kyoto Japan) for certain hours. Based on the values obtained, a growth curve was drawn and the resistance of the strain was assessed.

#### Resistance to the Gastric Environment

Acid and bile tolerance of LAB strain was examined by modifying the method determined by Lim & Im [28]. In order to determine the resistance of lactic acid bacteria in the stomach environment, centrifugation was applied at 5000 rpm for 15 min at room temperature to separate the *L. brevis* cells grown in MRS medium at 37 °C overnight. The cells were then washed twice with 0.85% physiological saline and resuspended in physiological saline. Then, 1% of the culture was inoculated into the MRS medium, without adjusting the pH and not adding pepsin for control purposes. Likewise, 1% of the culture was inoculated into 10 mL culture medium containing 3 mg/mL pepsin and adjusted to pH 2.5 with 5 N HCl. These processes were carried out in a glass tube and left to incubate at 37 °C for 2 h without shaking. For the viable cell count of the culture, three-parallel cultivation was performed on MRS agar medium after incubation (10^–4^ dilution) and after washing culture (10^–8^ dilution) with the spot method. Viable cell counts were made after overnight incubation at 37 °C.

#### Cell Surface Hydrophobicity

Hydrophobicity is defined as the ability of microorganisms to adhere to hydrocarbons [29]. To harvest *L. brevis* cells grown overnight at 37 °C in MRS broth, the culture was centrifuged at 5000 rpm for 15 min at room temperature, and the bacterial pellet collected during the stationary growth phase was washed twice with sterile saline solution (0.85% NaCl). They were then resuspended in phosphate buffer solution (PBS, pH: 7.2) until the optical density (at 600 nm) reached 0.6–0.7 (5 mL) (PBS containing 108 cfu/mL bacteria) (A_0_). Then, 1 mL of hydrocarbon was added to 3 mL of the washed cell suspension. After 10 min of pre-incubation at 23 °C, the 2-phase system was mixed for 2 min. It was then incubated at 23 °C for 20 min. The aqueous phase was carefully separated and absorbance was measured at 600 nm (A_1_). In the analysis, n-hexane was used as the hydrocarbon. Cell surface hydrophobicity was calculated according to Eq. (1) given below.

1 $$\text{cell surface hydrophobicity}(\mathrm{H\%})=(1-\frac{{\mathrm{A}}_{1}}{{\mathrm{A}}_{0}})\times 100$$

 

#### Antibacterial Activity

The antimicrobial spectrum of *Levilactobacillus brevis* AK19B was evaluated against Escherichia coli BC 1402, Bacillus cereus BC 6830, Salmonella typhimurium RSSK 95091, Yersinia enterocolitica ATCC 27729, and Staphylococcus aureus ATCC 25923 using the agar spot test method described by Schillinger and Lücke [30].

#### Antibiotic Resistance

The *L. brevis* strain was cultivated overnight at 37 °C to reach approximately 10⁷ CFU/mL. Then, 100 µL of the culture was spread onto MRS agar plates. Antibiotic susceptibility was tested using commercial antibiotic discs (Oxoid Antimicrobial Susceptibility Test Discs), including vancomycin, streptomycin, ampicillin, erythromycin, tetracycline, penicillin G, kanamycin, oxacillin, gentamicin, and chloramphenicol. Plates were incubated at 37 °C for 24 h, and inhibition zone diameters were recorded to determine the resistance profile of the strain.

#### Antioxidant Activity

To assess antioxidant activity, the LAB strain was cultured at 37 °C for 24 h. Then, 1 mL of 0.2 mM DPPH (2,2-diphenyl-1-picrylhydrazyl) solution—prepared in methanol—was added to 1 mL of the cultured isolate in MRS broth. For the control, distilled water was used instead of the sample. After vortexing, the mixtures were incubated in the dark at room temperature for 30 min. The samples were then centrifuged at 1400 × g for 10 min, and absorbance was measured at 517 nm against pure methanol. The percentage of DPPH radical scavenging activity was calculated accordingly.

#### Phytase Activity

Phytase activity was determined following the method of [31], with slight modifications. A 10 mL culture of *L. brevis* grown in MRS broth was centrifuged at 5000 rpm for 15 min at 4 °C. The supernatant (crude enzyme solution) was separated from the cell pellet. For the reaction, four test tubes and one blank were prepared. To each test tube, 900 µL of 2 mM sodium phytate solution in 0.1 M Tris–HCl buffer (pH 7.0) was added as substrate. For the blank, 750 µL of trichloroacetic acid (TCA) was added instead. All tubes were pre-incubated at 37 °C for 5 min. Then, 100 µL of enzyme solution was added to each sample tube, and 100 µL of Tris–HCl buffer was added to the blank. After incubation at 37 °C for 30 min, 750 µL of TCA was added to the test tubes, and 900 µL of substrate solution to the blank, followed by thorough mixing. Subsequently, 1500 µL of 2.5% ammonium molybdate prepared in 5.5% sulfuric acid and 1500 µL of 2.5% ferrous sulfate were added as color reagents. Absorbance was measured at 700 nm against the blank. Phytase activity was expressed in milliunits (mU), defined as the amount of enzyme that liberates 1 nmol of inorganic phosphate per minute under the assay conditions. Enzyme activity was calculated in milliunits (mU) and expressed as the amount of enzyme that released 1 nmol of phosphate per minute under standard experimental conditions.

Additionally, all biochemical measurements, including GABA quantification, antioxidant activity, and enzyme assays, were performed in triplicate (*n* = 3), and results are presented as mean ± standard deviation.

### Optimization of GABA Production

#### Experimental Design

The purpose of using the response surface methodology (RSM) in this study was to optimize GABA production while minimizing the number of experimental runs and shortening the time required to determine the effects of key parameters. The aim was to develop a statistical model describing the relationship between GABA yield and selected production factors. Experimental design and statistical analyses were carried out using the Design-Expert 12 software (Stat-Ease Inc., Version 12.0, Minneapolis, USA). The Box–Behnken design was employed to evaluate the effects of four independent variables: MSG concentration (mM), pH, temperature (°C), and NaCl concentration (mM), on the GABA production (mg/mL) as measured by HPLC ( Although preliminary experiments revealed semi-optimal pH and temperature conditions for cell growth, these conditions were not directly used in the optimization study. This is because optimal bacterial growth does not always correlate with maximum metabolite production. In particular, previous studies have demonstrated that sub-optimal or mildly acidic conditions may enhance GABA biosynthesis by increasing glutamate decarboxylase (GAD) activity in lactic acid bacteria [32, 33]. Therefore, the selected design space intentionally included lower pH values (e.g., pH 4) and broader temperature ranges to account for this metabolic behavior. All experimental runs were conducted in 15 mL sterile screw-capped tubes containing 10 mL of MRS broth supplemented with the specified MSG and NaCl concentrations. The initial pH was adjusted using 1 M NaOH or HCl prior to autoclaving. A 1% (v/v) inoculum of a 24-h *Levilactobacillus brevis* AK19B pre-culture (~ 10⁷ CFU/mL) was transferred into each tube. Incubation was performed at the corresponding temperature (based on the experimental design) without shaking for 72 h. After incubation, 2 mL of culture was withdrawn, centrifuged at 5000 rpm for 10 min, and the supernatant was filtered through a 0.22 µm membrane prior to HPLC analysis. Each experimental point in the Box–Behnken design was conducted with two independent replicates. In the statistical analysis, a second-order polynomial regression model was fitted to the data using Eq. 2: This limitation has been acknowledged, and the need for future biological replicates is now emphasized in the manuscript to strengthen statistical robustness.

2 $$Y={\beta }_{0}+\sum {\beta }_{i}{X}_{i}+\sum {\beta }_{ij}{X}_{i}{X}_{j}+\sum {\beta }_{ii}{X}_{i}^{2}$$

For optimization of the independent variables, conditions where the GABA production value is maximized and the desirability value is 1 were chosen. The lower and upper limit values of the independent variables used for optimization of the GABA production process and the experimental design used in the study are given in Table 1.

**Table 1:** Experimental desgin parameters, conditions and ANOVA results of GABA production

Independent variables		pH	MSG concentration (mM)	Temperature (C°)	NaCl Concentration				
Unit			mM	C°	mM				
Symbol	Codded	X_1_	X_2_	X_3_	X_4_				
Factor level	-1	4	100	25	0				
	0	6	200	32.5	2				
	1	8	300	40	4				
		**Independent Variables**			**Response**				
Run	**pH**	**MSG concentration (mM)**	**Temperature (C°)**	**NaCl Concentration (mM)**	**GABA Production(mg/ml) **				
2	6	100	32.5	0	14.361				
3	8	300	32.5	2	20.862				
4	8	100	32.5	2	16.647				
5	6	200	25.0	0	28.180				
6	6	300	32.5	4	43.762				
7	6	300	32.5	0	34.208				
8	6	300	40.0	2	10.368				
9	6	100	40.0	2	5.511				
10	4	200	32.5	4	30.414				
11	4	300	32.5	2	38.104				
12	6	300	25.0	2	31.183				
13	4	200	40.0	2	0.022				
14	6	200	32.5	2	29.376				
16	4	200	25.0	2	32.979				
17	8	200	40.0	2	0.0111				
18	8	200	32.5	4	17.603				
19	6	200	40.0	4	10.574				
20	8	200	32.5	0	23.525				
21	6	100	32.5	4	18.259				
22	6	200	25.0	4	35.805				
23	6	200	32.5	2	17.440				
24	4	200	32.5	0	24.509				
25	8	200	25.0	2	19.686				
26	6	200	32.5	2	29.291				
27	6	200	32.5	2	34.313				
28	6	200	40.0	0	6.597				
RSM model statistics and ANOVA analysis of GABA production									
Model	**SD**	**p-value**	R^2^	**R**^2^**adj**	**R**^2^**pred**	**LOF **	**LOF **	**press**	
						F value	p-value		
Lineer	7.32	<0.0001	0.6552	0.5952	0.4983	1.05	0.5671	1791.39	
2FI	7.91	0.8354	0.7024	0.5274	0.1909	1.26	0.4821	2888.84	
Quadratic*	5.19	0.0039	0.902	0.7965	0.6076	0.3802	0.8923	1400.99	suggested
Cubic	5.78	0.6998	0.9532	0.7471	0.4022	0.1256	0.8864	2134.53	aliased
Model	27.61		3220.49	14	230.3	8.55	0.0002	significant	
X_1_ (pH)	-3.81		174.27	1	174.27	6.47	0.0244		
X_2_ (MSG concentration)	7.27		633.55	1	633.55	23.54	0.0003		
X_3_ (Temperature)	-11.1		1479.21	1	1479.21	54.96	<0.0001		
X_4_ (NaCl concentration)	2.09		52.24	1	52.24	1.94	0.187		
X_1_*X_2_	-3.96		62.83	1	62.83	2.33	0.1505		
X_1_*X_3_	3.32		44.1	1	44.1	1.64	0.2229		
X_2_*X_3_	-1.96		15.38	1	15.38	0.5715	0.4631		
X_2_*X_4_	1.41		8	1	8	0.2971	0.5949		
X3*X_4_	-0.9119		3.33	1	3.33	0.1236	0.7308		
X_1_^2^	-4.32		112.13	1	112.13	4.17	0.0621		
X_2_^2^	-0.9002		4.86	1	4.86	0.1806	0.6778		
X_3_^2^	-9.7		564.33	1	564.33	20.97	0.0005		
X_4_^2^	1.35		10.96	1	10.96	0.4073	0.5344		
Residual			349.92	13	26.92				
Lack of fit			195.6	10	19.56	0.3802	0.8923	insignificant	
Error			154.32	3	51.44				

For modeling, the degree of desirability (D) was calculated for the optimization process by evaluating the responses obtained. Levels of independent variables were determined in conditions where GABA production was maximized and D = 1. Design-Expert software (Stat-Ease Inc. Version 12.0 Minneapolis, USA) was used for modeling, optimizing and providing 3D graphics by analyzing empirical data.

## Results and Discussion

### Isolates Capable of Producing GABA

**Fig. 1:**
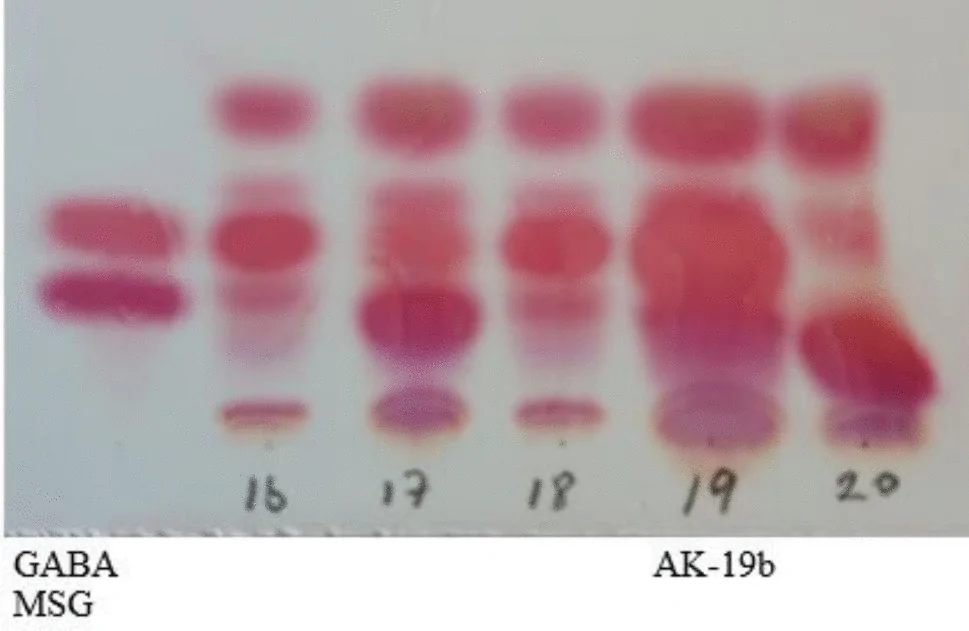
TLC image showing *L. brevis* AK19B GABA production

GABA production abilities of 30 lactic acid bacteria (LAB) isolates from sourdough were initially screened using thin-layer chromatography (TLC) following their cultivation in MRS medium supplemented with 53 mM MSG for 96 h at 37 °C. All isolates were preliminarily screened for GABA production via TLC. Among them, strain AK19B showed the most prominent GABA spot intensity. Following this, quantitative determination by HPLC was performed, which confirmed that AK19B produced the highest GABA levels among tested isolates (data not shown). Significant GABA production were visually presented in Fig. 1 to facilitate clear comparison and interpretation. This isolate was subsequently subjected to genotypic identification through 16S rRNA gene sequencing.

### Identification of Lactic Acid Bacteria

Genotypic identification of the AK19B isolate with high GABA production ability was performed. The strain identified as *Lactobacillus brevis* AK19B (Accession number: MZ636922) was determined to be gram positive, catalase negative. Bacteria were stored at −80 °C in 40% glycerol for further diagnostic testing.

### Growth at Different Ph

Deamidation occurring in sourdough contributes to the growth of lactic acid bacteria such as *Lactobacillius reuteri* in an acidic environment [34]. The growth of bacteria in MRS broth with pH 4, pH 5 and pH 8 is given in Table 2. Absorbance results were rated as poor, good, and very good. Based on the results obtained, the growth of *L. brevis* AK19B strain was evaluated as very good at pH 5 and pH 8, but its growth remained weak at pH 4.

**Table 2:** Some properties of *L. brevis* AK19B

Growth conditions*									
pH 8	**pH 5**	**pH 4**	**4 ℃**	**25 ℃**	**45 ℃**	**7% NaCI**	**10% NaCI**	**15% NaCI**	
VG^a^	VG^a^	P^b^	-^c^	VG^a^	P^b^	VG ^a^	P^b^	P^b^	
Antimicrobial activity**									
P12	**P60**	**P78**	**P91**						
+++^ab^	++^b^	+++^ab^	+++^a^						
Antibiyotic resistance (Zone diameter)***									
VA (30 µg)	**S (10 µg)**	**AMP (10 µg)**	**E (15 µg)**	**C (30 µg)**	**CN (10 µg)**	**OX (1 µg)**	**K (30 µg)**	**P (10 µg)**	**TE (30 µg)**
-	2±0^b^	14±0^a^	18±2.12^a^	16±1.41^a^	-	-	2±0^b^	15±2.12^a^	3±0^b^
Antioxidant activity									
DPPH Inhibition%									

65.06±0.85

*P; Optical density at 600 nm (OD)≤ 1,0 G: OD= 1,1-1,5, VG: OD≥ 1,5*(P12: *E. coli* BC 1402, P60: *B. cereus* BC 6830, P78: *S. typhimurium* RSSK 95091, P91: *Y. enterocolitica* ATCC 27729, P94: *S. aureus* ATCC 25923)

**(-): None; Zone diameters are (+): 0-14 mm; (++): 14-18 mm; (+++): >18 mm

***Vancomycin (VA), Streptomycin (S), Ampicilin (AMP), Erythromycin €, Chloromphenicol ©, Gentamicin (CN), Oxacilin (OX), Kanamycin (K), Penicilin G (P), Tetracycline (TE) a-c. Different lowercase letters within the same row indicate statistically significant differences (*p* < 0.05)

### Growth at Different Temperatures

The growth of bacteria in MRS broth for 7 days at 4 °C, 2 days at 25 °C and 3 days at 45 °C is given in Table 2. Absorbance results were evaluated as poor, good, and very good. The growth of the bacteria in 2 days at 25 °C was evaluated as very good, while the growth was weak for other temperatures.

The growth of bacteria occurs in parallel to the increase in temperature and increases up to a certain temperature. This relationship between temperature and bacterial growth is similar for GABA production. In a study [35], it was concluded that the GABA production of *L. brevis* NCL912 reached the maximum level at 35 °C, and bacterial growth and GABA production were not observed at 45 °C. This result is parallel with the result obtained from the study. High temperature was determined to affect the growth of *L. brevis* AK19B negatively and it is thought that it will negatively affect GABA production at high temperature.

### Thermotolerant Capacity

The resistance of strains to heat treatment is important for products that are heat treated after fermentation [36]. In fact, the survival of lactic acid bacteria after heat treatment may be an indication that thermotolerant lactic acid bacteria can be used as a bioprotective culture to ensure microbial safety, as it can extend the shelf life of cooked products [37, 38].

Bacteria cannot grow at extremely high temperatures such as 70 ℃, as high temperature negatively affects bacterial growth (although it differs between bacterial species). The weak growth of *L. brevis* AK19B at 45 ℃ suggests that the bacteria cannot grow at a temperature of 70 ℃. As a result of the analysis, the bacteria do not have the ability to grow at high temperatures (70 ℃); that is, they do not have thermotolerant properties.

### Growth at High Salt Concentrations

The salt content of the source from which the bacterium is isolated can give an idea about the growth of the bacterium in a high salt environment. *L. brevis* AK19B strain was developed in MRS broth containing 7%, 10% and 15% NaCl. Based on the results obtained, the growth of bacteria in the medium containing 7% NaCl showed good improvement compared to the growth in the medium containing 10% NaCl and 15% NaCl. Based on the results, environments containing high concentrations of salt have a negative effect on the growth of *L. brevis* AK19B (Table 2).

### Resistance to Bile Salt

**Fig. 2:**
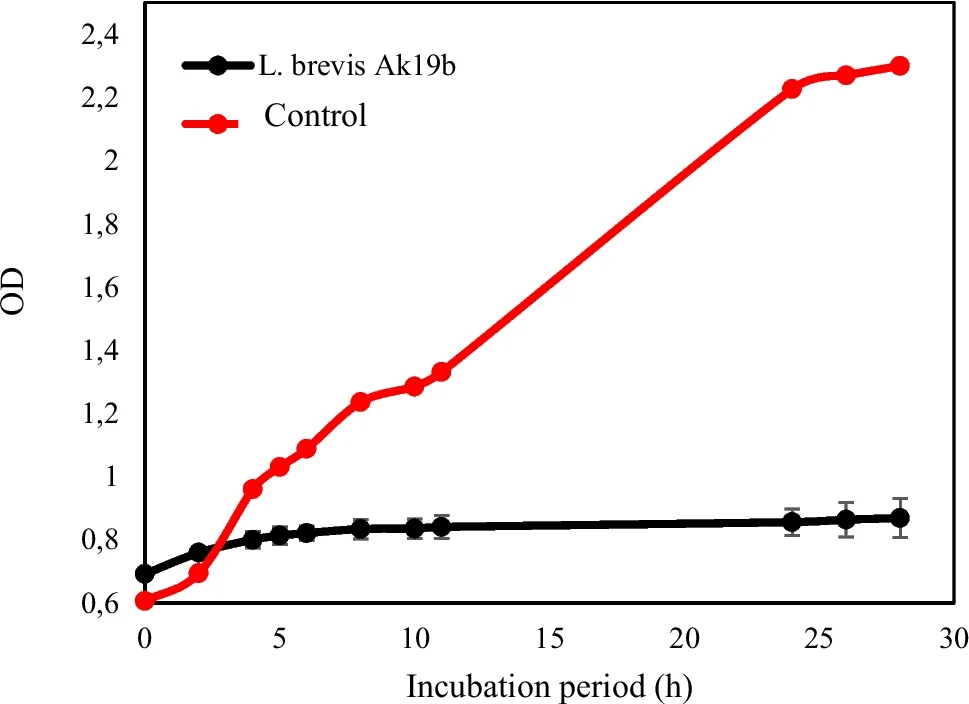
*L. brevis* AK19B growth in the presence of bile salts

Bile is synthesized in the liver, stored in the gallbladder, and secreted into the small intestine to emulsify and dissolve fat when the body needs it [39, 40, 41–43]. Bile salts are formed as a result of the conjugation of bile acids. They have an important detergent effect, ensuring the excretion of cholesterol from the liver. Bile acids also ensure that digestive enzymes work at optimum levels [44]. In order for effects to be seen, probiotic bacteria must be able to pass through the gastrointestinal tract, maintain their vitality in acidic environments (pH 2.5–3.5) and in digestive system conditions containing 0.3% bile salt [45, 62]. *L. brevis* AK19B, developed in MRS broth containing 0.3% bile salt and pH 4, continued its development up to a certain level as can be seen in Fig. 2. In studies with Lactobacillius cultures, the growth of cultures slowed down at 0.3% bile salt concentrations [41, 42].

### Resistance to the Stomach Environment

In the selection of strains with probiotic character, acid tolerance is a key feature to ensure the viability and functionality of the strain. For this reason, various models were developed to simulate the gastrointestinal system, and incubation for 1–4 h was performed in enzymatic environments with a pH of 1.5–3 [43]. The tolerance of *L. brevis* AK19B strain in low pH and enzymatic media was determined. As a result of 2-h incubation at pH 2.5 in enzymatic medium containing 3 mg/mL pepsin, high viable count was determined (2 × 10^3^ cfu/mL) (viable cell count before incubation was 1.8 × 10^9^ cfu/mL) In many studies, lactic acid bacteria and bifidobacteria with probiotic properties had acid tolerance and can adhere to the intestines [46–48]. This result is in agreement with other studies in the literature [49, 62], and shows that *L. brevis* AK19B has the ability to adhere to the cell wall in the stomach and can survive bile salts. In addition, it can be said that it has probiotic character based on these results.

### Cell Surface Hydrophobicity

The method used to determine the ability to adhere to hydrocarbons gives the hydrophobic properties of microorganisms. This feature has great importance as it modulates the immune system, increases the healing of damaged mucosa and reduces colonization by pathogenic microorganisms. Hydrophobicity, which is one of the properties that is effective on the colonization of probiotic bacteria in the intestine, is affected by the surface properties of the microorganism. Hydrophobic components that affect adhesion to the intestinal surface are structures such as adhesive or polysaccharide, teichoic acid and fatty acids. Covalent interaction occurs between the carbohydrate extensions of glycoproteins and glycolipids in the intestinal epithelium and the hydrophobic structures on the surfaces of microorganisms [50]. Many factors such as the growth environment of the bacteria, low pH, carbohydrates in the bacterial cell and the treatment of bacterial cells with protease affect the adhesion of bacteria [51].

In our study, the surface hydrophobicity of *L. brevis* AK19B was measured at 9.87 ± 2.65%, which places it within the hydrophilic category according to Grajek et al. [50], who define hydrophilicity as values below 20%. When compared to previous reports, this value is somewhat higher than that observed for certain *L. brevis* strains [52], yet considerably lower than the notably hydrophobic *L. brevis* KU200019 strain, which exhibited hydrophobicity exceeding 50% as reported by Kariyawasam et al. [53]. These results suggest that while AK19B is predominantly hydrophilic, it shows modestly greater surface affinity towards hydrophobic interactions compared to some related strains.

### Antimicrobial Spectrum

As a result of the analysis, *L. brevis* AK19B had a suppressive effect on the growth of pathogenic microorganisms. As shown in Table 2, AK19B was determined to have antimicrobial activity against *E. coli* BC 1402, *B. cereus* BC 6830, *Y. enterocolitica* ATCC 27729 and *S. typhimurium* RSSK 95091. Jang et al. [52] reported that *L. brevis* KU15153 isolate had significant antibacterial activity against *E. coli* ATCC 25922, *Listeria monocytogene*s ATCC 15313, *S. typhimurium* P99 and *Staphylococcus aureus* KCCM 11335.

The antimicrobial activity is due to the production of metabolites such as acetic acid, lactic acid, diacetyl and bacteriocin [52, 54, 55].

### Antioxidative Activity

With the metabolism of lactic acid bacteria in sourdough, they can create lipid oxidation and a strong antioxidative effect during fermentation [56]. Antioxidant activities of LAB strains play an important role in protection from free radicals [52]. Some lactobacillus species have antioxidant activity and can reduce the risk of accumulation of reactive oxygen species in the body [57]. The radical scavenging activity of *L. brevis* LAP2 increased depending on the concentration (18.8–68.68% in 10^8^–10^9^ cfu mL^−1^) [58]. It was determined that it had a very low radical scavenging activity with values of 3 [59]. The difference in antioxidant activity varies depending on the metabolite production of the bacteria, enzyme, bacterial cell wall composition and is strain-specific [52].

The antioxidant activity of *L. brevis* AK19B was calculated as 65.06 ± 0.85%. Behnahani et al. [60] determined the antioxidant activity of *L. brevis* A3 isolate to be 59.67 ± 0.79%. When compared with the antioxidant activity of the *L. brevis* A3 isolate, *L. brevis* AK-19 had higher antioxidant activity.

### Phytase Enzyme Activity

Phytic acid (myo-inositol-1,2,3,4,5,6-hexakis dihydrogen phosphate) constitutes approximately 1–4% of cereal grains and plays a dual role as a source of phosphorus and myo-inositol, yet its chelating nature negatively affects nutrient absorption due to binding with minerals and amino acids [61, 62]. In our study, the absorbance at 700 nm for *L. brevis* AK19B was measured at 0.790, which translates—via the standard curve and activity formula—to a phytase activity of **5.31 ± 0.17 mU/mL**. The extracellular phytase activity of AK19B supports its potential use as a starter culture, especially since intracellular enzyme presence may suggest additional functional contributions upon cell lysis [62].

The phytase activity of *L. brevis* AK19B (5.31 mU/mL) may offer more than nutritional improvement it may indirectly support enhanced GABA production. By hydrolyzing phytic acid a widespread anti-nutritional factor in cereals the enzyme releases minerals like Mg^2^⁺ and phosphate, which are cofactors necessary for PLP-dependent glutamate decarboxylase, central to GABA biosynthesis [63]. Additionally, phenolic and antioxidant systems may reduce oxidative stress during fermentation, protecting both cells and enzymes, thereby supporting sustained metabolic activity. Altogether, these traits position AK19B not only as a GABA producer but also as a functional starter with significant advantages for mineral bioavailability and fermentation resilience.

### Experimental Design

In the optimization of GABA production, statistical analyses were performed using the responses provided by the Box-Behnken experimental matrix given in Table 1. In the modeling phase, the response surface method statistics and ANOVA results of some models are given in Table 1.

**Fig. 3:**
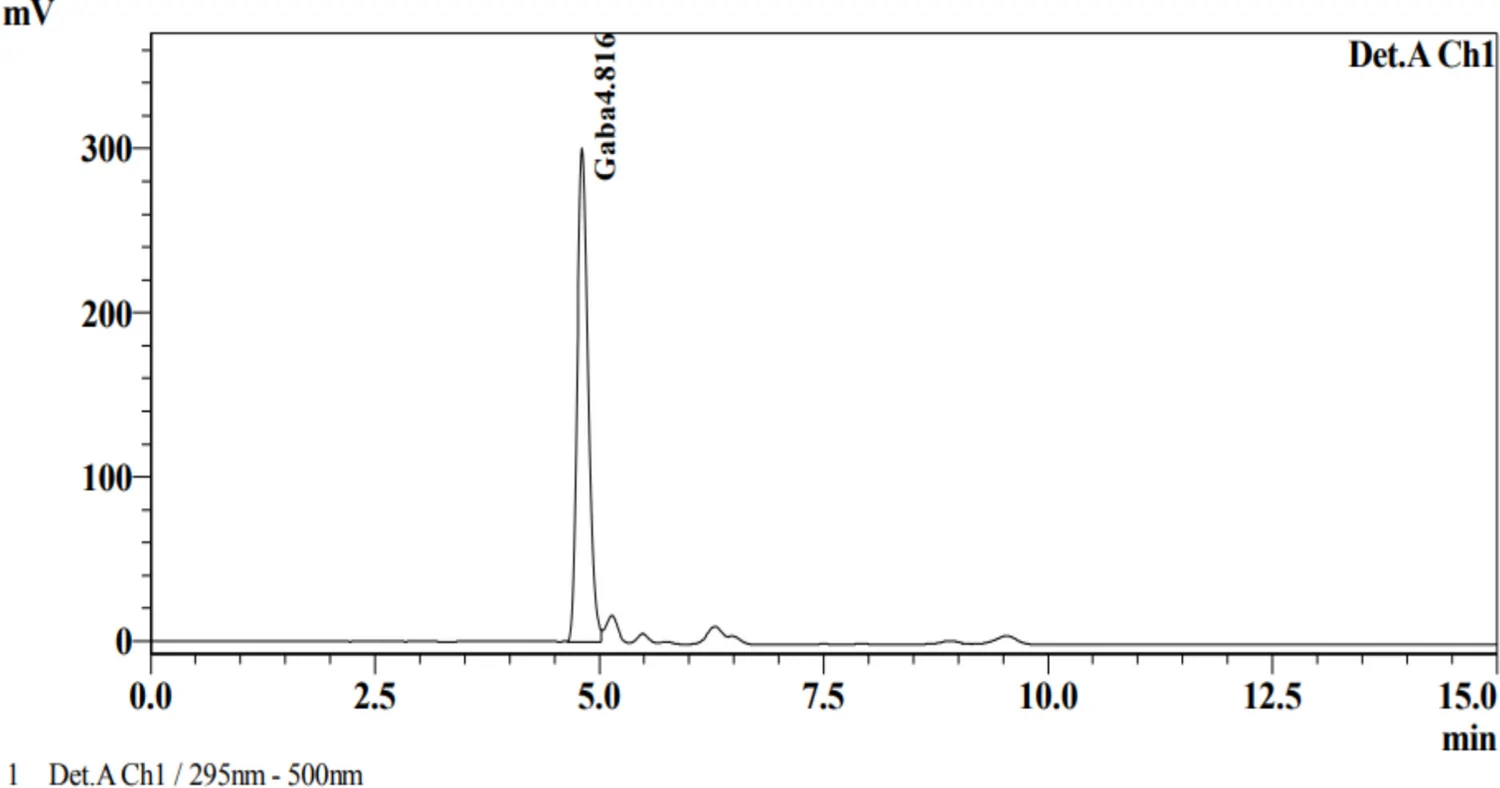
An HPLC chromatogram of GABA production in modified MRS medium by *Levilactobacillus brevis* AK19B (pH 6, MSG 200 mM, 32.5 °C, NaCl 2 mM)

The experimental design matrix determined for the test conditions for GABA production is given in Table 1. The conditions used in the experimental design matrix vary between 4–8 for pH, 25–40 ºC incubation temperature, 100–300 mM for MSG concentration and 0–4 mM for NaCl concentration. The effects of independent variables on GABA production were determined with different experiments. In experiment 6, the highest level of GABA production (43.762 mg/mL) was detected, while the lowest GABA production was determined at pH 8 in experiment 6, with pH 6, MSG concentration 300 mM, temperature 32.5, and NaCl content 4 mM. GABA production was determined at a concentration of 0.0110 mg GABA/mL under conditions with 200 mM MSG, 2 mM NaCl and an incubation temperature of 40 ºC. A representative HPLC chromatogram for GABA detection under typical experimental conditions is shown in Fig. 3.

When the ANOVA and RSM model statistics in Table 1. are evaluated, the quadratic model had the best prediction level compared to the linear, 2FI and cubic models. However, the determination coefficient for the cubic model, which was evaluated by the Design-Expert software for the GABA production response, was higher (0.9532) compared to the quadratic model, and the p value of 0.6998 rendered this model insignificant. The quadratic model had high coefficient of determination (R^2^), corrected coefficient of determination (R^2^adj) and estimated coefficient of determination (R^2^pre), and the p-value it provides was considerably lower than other models. For this reason, the equation obtained for GABA production using the quadratic model is given in Eq. 3.

3 $$\begin{array}{l} {Y\left(GABA concentration\right)}={\mathrm{27.61}-\mathrm{3.81}X}_{1}\\+{\mathrm{7.27}X}_{2}{-\mathrm{11.10}X}_{3}{+\mathrm{2.09}X}_{4}-{\mathrm{3.96}X}_{1}*{X}_{2}{+\mathrm{3.32}X}_{1}\\*{X}_{3 }-\mathrm{2.96}{X}_{1}*{X}_{4 }-{\mathrm{1.96}X}_{2}*{X}_{3 }+\mathrm{1.41}{X}_{2}\\*{X}_{4 }-\mathrm{0.9119}{X}_{3}*{X}_{4 }-\mathrm{4.32}{X}_{1}^{2}-\mathrm{0.9002}{X}_{2}^{2}-\mathrm{9.70}{X}_{3}^{2}+\mathrm{1.35}{X}_{4}^{2} \end{array}$$

Studies in which the quadratic model is preferred for optimization of GABA production with lactic acid bacteria are quite common [64, 65].

The ANOVA values given in Table 1. show that the Box–Behnken model obtained from the Response Surface Methodology (RSM) analysis was found to be statistically significant for GABA production. According to the ANOVA results, the quadratic model was recommended as the most suitable model (*p* = 0.0039). The model’s R^2^ value is 0.902, the adjusted R^2^ value is 0.7965, and the predictable R^2^ value is 0.6076, indicating that the model explains a large portion of the variance in the variables. The model’s Lack-of-Fit value was insignificant (F = 0.3802; *p* = 0.8923), indicating that the model fits the data well. When examining the effects of the variables, temperature (X3) was determined to be the strongest and most significant factor (*p* < 0.0001), followed by MSG concentration (X2) and pH (X1). The effect of NaCl (X4) was not found to be significant. Furthermore, the significance of the second-order term X3^2^ (*p* = 0.0005) indicates that the temperature variable has a curvilinear effect on GABA production. These results confirm that the established quadratic model reliably predicts GABA production and that the Box–Behnken design is suitable for the study.

**Fig. 4:**
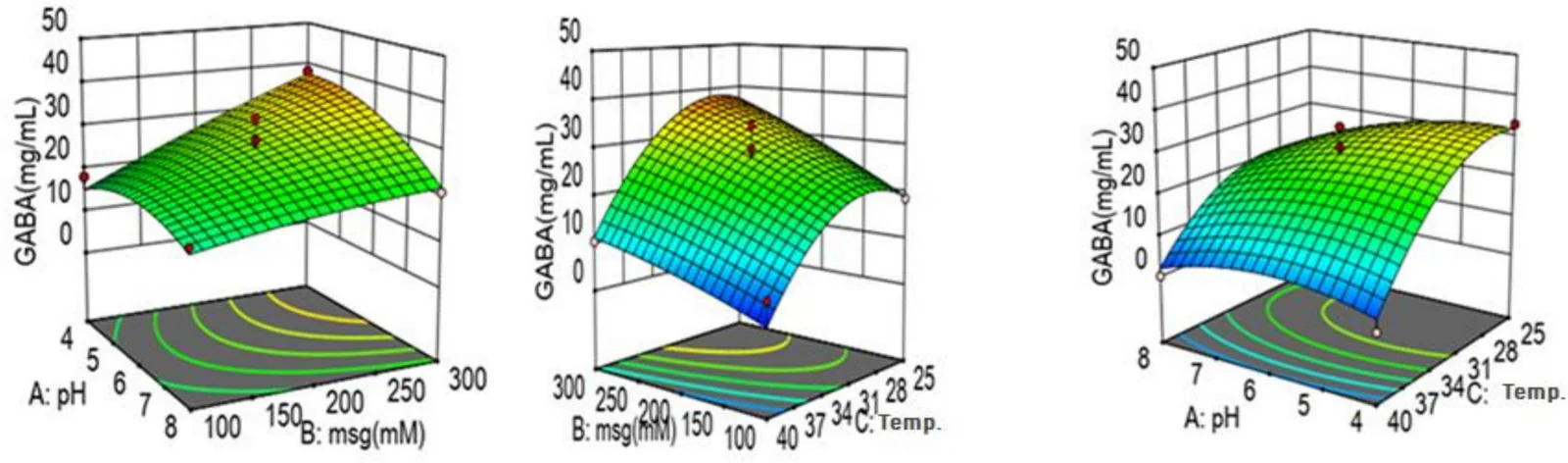
The effect of temperature (temp.), pH, NaCl and MSG concentration on GABA production **A** Three-dimensional interaction plot of MSG* pH data (temperature = 32.5ºC NaCl = 2mM) **B** Three-dimensional interaction plot of temperature*MSG data (pH = 6, NaCl = 2mM) **C** Three-dimensional interaction plot of pH*temperature data (MSG = 300mM, NaCl = 2mM)

The 3D and contour graphics in Fig. 4 show the effects of the independent variables. When the effect of temperature on GABA production is examined, GABA production increases with the decrease in pH. At low pH, when the temperature is high and the MSG concentration is low, GABA production also decreases, while the effect of MSG and NaCl concentrations varies.

The gadC gene, which is effective in the production of GABA identified in *L. brevis*, is more active above pH 5.6 [66]. Organic acids such as lactic acid produced by bacteria during 96 h of incubation of *L. brevis* AK19B reduce the pH of the environment to an acidity below 5.6, and accordingly, GABA production decreases. Cataldo, Villegas, Savoy de Giori, Saavedra, & Hebert [67] adjusted the initial pH between 5.8–7.25 and *L. brevis* CRL2013 was not affected. But at pH values below 5.8 (pH 4), they found a sharp decrease in the level of GABA. This result obtained with *L. brevis* AK19B reveals the dependence of GABA production on pH. The variation in NaCl concentration for GABA production with *L. brevis* AK19B strain may be related to the salt content of the source from which the bacteria was isolated. The GABA production of *L. brevis* AK19B under optimized conditions was found to be 43.85 mg/mL, which is slightly higher than that reported for *L. brevis* K203 (42 mg/mL) by Binh et al. [68]. In comparison, Lactococcus lactis NCDO 2118 has been reported to produce up to 50.8 mM (approx. 5.3 mg/mL) GABA under optimal conditions [17], while Lactobacillus plantarum Taj-Apis362 reached approximately 26.4 mg/mL [64]. Other strains such as Streptococcus salivarius subsp. thermophilus Y2 have demonstrated lower yields (around 6 mg/mL) [69]. These comparisons suggest that *L. brevis* AK19B is among the top-performing LAB strains reported to date in terms of GABA yield. Furthermore, its combined probiotic potential and phytase activity reinforce its applicability in functional food fermentation.

As in this study, the relationship between the growth of bacteria and temperature also affects GABA production. Cataldo et al. [15] investigated the effects of culture conditions on *L. brevis* CRL 2013 GABA production and found that the highest GABA (265 mM) yield was obtained at 30 °C and 37 °C within temperatures ranging from 20 to 45 °C. They reported that GABA production decreased significantly with increasing temperature. Li, Qui, Huang, & Cao [35] in their study with *L. brevis* NCL912 observed no bacterial growth and GABA production at 45 °C. Based on the results obtained, high cell density as well as appropriate temperature values are effective for GABA production at maximum efficiency.

The desirability value was taken into account for the optimization of the independent variables. For this purpose, pH 4.01–5.73 with D = 1 value, MSG concentration 231.43–299.96 mM, temperature 25.01–30.82 ºC and NaCl concentration 1.89–4 mM were determined. A total of one hundred possible solution combinations were evaluated during the optimization process using the Design-Expert software.

### Validation of the Model

**Fig. 5:**
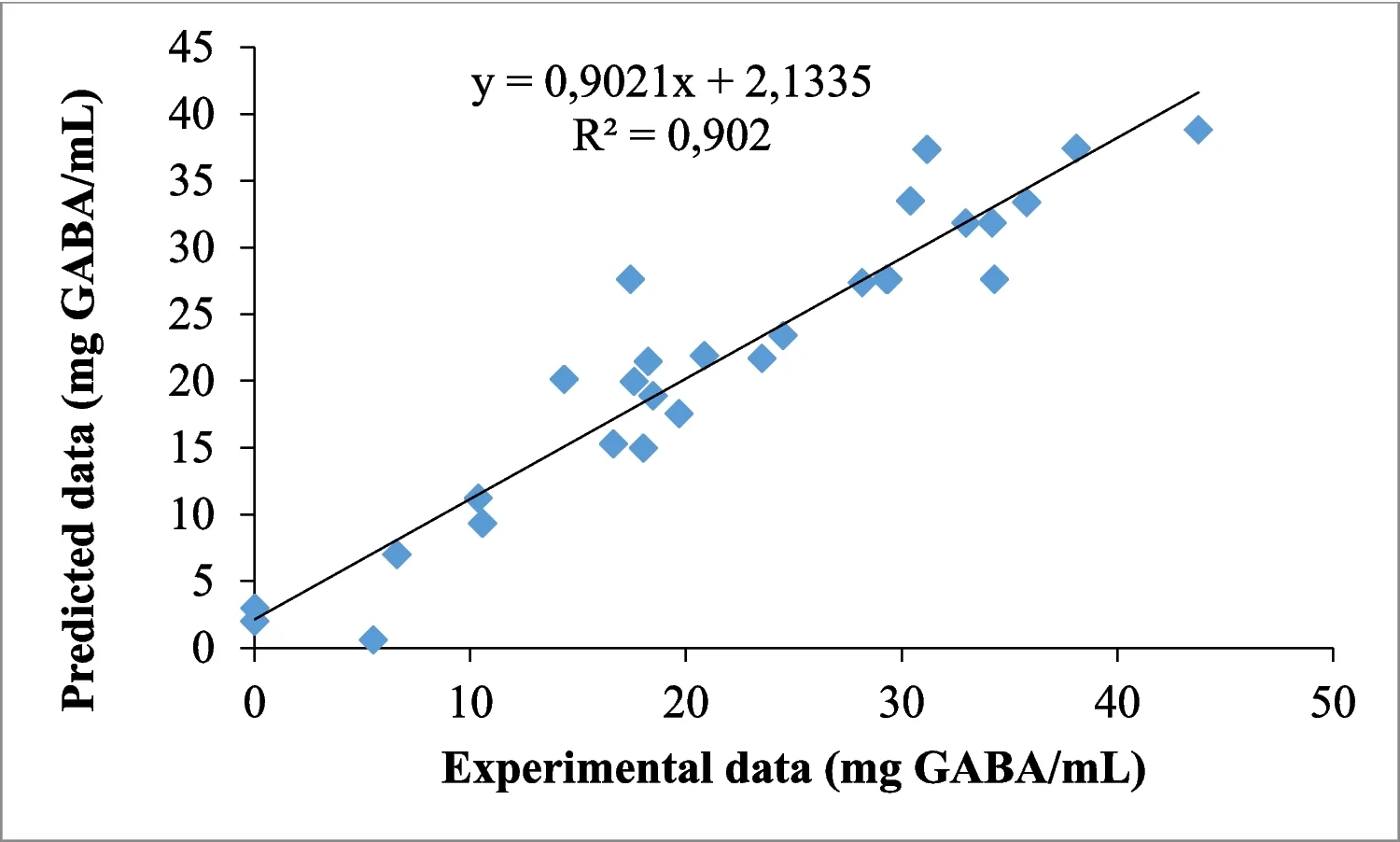
The compatibility of the model obtained with the optimization study of GABA production of *L. brevis* AK-19b strain with the experimental data

The compatibility of the post-production data, using the model obtained from the optimization of *L. brevis* AK19B strain and the GABA production conditions, is shown in the graph in Fig. 5. The R^2^ value of the model belonging to the experimental data and the estimated data is close to 1.

In this optimization study, in order to maximize the proposed GABA production, GABA production was estimated as 43.85 mg/mL under the predicted optimum conditions (pH 5.14, MSG concentration 297 mM, temperature 26 °C, and NaCl concentration 2.82 mM) with a desirability (D) value of 1.00. Based on the model’s residual standard deviation (5.19 mg/mL), the 95% prediction interval was calculated as approximately 33.03–54.67 mg/mL.

In this study, our main focus was to assess and optimize the ability of Levilactobacillus brevis AK19B to produce GABA under controlled laboratory conditions, providing a strong foundation for its potential industrial application. However, we did not delve deeply into the complex metabolic pathways or genetic factors particularly those related to the glutamate decarboxylase (GAD) system that drive GABA biosynthesis. A deeper understanding of these mechanisms could be achieved in future work through approaches such as gene expression analysis or proteomics. In addition, while the laboratory-scale optimization yielded promising GABA levels, important aspects related to industrial-scale production, including bioreactor configuration, process fine-tuning, and economic feasibility, were beyond the scope of the current study. Addressing these areas in future research will be crucial for translating the strain’s potential into a commercially viable GABA production process.

## Conclusion

The use of GABA-producing lactic acid bacteria represents a promising alternative and economically viable approach to increase the concentration of this compound in foods. Since Since *L. brevis* is one of the strains commonly found in fermented foods, it is crucial to use this strain to produce GABA efficiently. The lactic acid bacteria isolated from sourdough, with probiotic character and identified as *L. brevis* AK19B, are resistant to vancomycin, gentamicin and oxacillin antibiotics. In addition, it is important at this point that probiotic bacteria, which have a special function against acute infections caused by pathogens resistant to various drugs, show resistance to vancomycin. In order to optimize GABA production, the production parameters to be used in the optimization study were determined and production was carried out accordingly. When the effect of temperature on GABA production was examined, GABA production increased at low pH values. GABA production decreased when the temperature is high and the MSG concentration is low. The effect of MSG and NaCl concentration on production varied. As a result of the production carried out under optimum conditions in the study, 43.85 mg/mL GABA production was estimated with *L. brevis* AK19B. In conclusion, in this study, the techno-functional properties of *L. brevis* AK19B isolate were identified and GABA production was optimized. These results will contribute to the understanding of the regulation of the GAD system in LAB and allow the development of GABA-enriched fermented products. In addition to acid and bile salt tolerance, this strain demonstrated significant antioxidant and antibacterial activity, further supporting its probiotic potential. Therefore, *L. brevis* AK19B can be used as a multifunctional starter culture that simultaneously enriches GABA content and promotes intestinal health. This dual functionality offers promising applications in the development of functional fermented foods, such as probiotic beverages, fermented cereals, and dairy alternatives aimed at stress modulation, antioxidant support, and gut health. This enhanced ability of *L. brevis* AK19B to synthesize GABA may open new perspectives in the production of GABA-enriched products.

## Data Availability

The data that support the findings of this study are available from the corresponding author upon reasonable request.
